# The *SAPALDIA* cohort: design and results in the field of respiratory and cardio-vascular diseases

**DOI:** 10.1186/1753-6561-7-S5-O5

**Published:** 2013-08-30

**Authors:** T Rochat

**Affiliations:** 1 Pulmonary Division, University Hospitals of Geneva, HUGE Geneva/SAPALDIA, Switzerland

## 

The SAPALDIA (Swiss Study on Air Pollution and Lung Disease in Adults) cohort involving eight geographical areas of Switzerland was initiated in 1991 for studying the effects of air pollution on the respiratory and cardiovascular health in adults. The study was initiated with a cross-sectional interview of 9651 subjects aged 18 to 60 years and more than 90% of them underwent lung function and atopy testing. More than 7000 of the subjects had bronchial reactivity tested by a methacholine challenge. The address histories of the participants were carefully followed up and a follow up assessment was done in 2002 (SAPALDIA 2). A second follow-up was done in 2010-11. In the 2002 follow-up, 8047 (83%) provided health information, 6528 persons underwent physical re-examination, and 6345 provided blood samples to establish an extensive blood, plasma, serum and DNA bank. In addition, 1813 subjects aged 50 or older participated in 24h-ECG Holter monitoring to provide detailed data on parameters of heart rate variability as an early marker of cardiac response to the environment. With the inclusion of cardiovascular endpoints, SAPALDIA is one of the first studies examining effects from long-term exposure to air pollution on cardiovascular health parameters as well as mutual influence between the respiratory and the cardiovascular system.

A paper validating the prediction model for individual exposure to PM10 particles was published and another paper published in the *New England Journal of Medicine* demonstrates that outdoor air pollution is associated with the evolution of individual lung function in adults. The SAPALDIA bio-bank has allowed scientific publications on the association between gene polymorphisms and the propensity to develop asthma, allergic diseases, or accelerated lung function decline with age. Ongoing studies are focusing on gene-environment interactions a crucial question to understand why some persons suffer more from the effect of air pollution than others.

The recent publications by the SAPALDIA team have addressed the key questions of the project: i) the impact of air pollution on respiratory and cardio-vascular health in a longitudinal perspective; ii) the role of genetic factors in modifying health effects from the environment, and iii) the complex interactions between respiratory and cardiovascular health. Further studies and refinements are necessary to address many unresolved issues. These require extensive collaborations, particularly in the fields of exposure assessment and genetic associations.

